# Effects of Fine Particulate Matter on Systemic Inflammatory Biomarkers in Elderly Patients with Chronic Obstructive Pulmonary Disease Versus the Healthy Elderly: A Pilot Study

**DOI:** 10.3390/ijms27146283

**Published:** 2026-07-15

**Authors:** Warawut Chaiwong, Chalerm Liwsrisakun, Pilaiporn Duangjit, Juthamas Inchai, Chaiwat Bumroongkit, Athavudh Deesomchok, Theerakorn Theerakittikul, Atikun Limsukon, Pattraporn Tajarernmuang, Nutchanok Niyatiwatchanchai, Konlawij Trongtrakul, Chittrawadee Chitchun, Nipon Chattipakorn, Siriporn C. Chattipakorn, Nattayaporn Apaijai, Chaicharn Pothirat

**Affiliations:** 1 Division of Pulmonary, Critical Care, and Allergy, Department of Internal Medicine, Faculty of Medicine, Chiang Mai University, Chiang Mai 50200, Thailand; warawut.chai@cmu.ac.th (W.C.); pilaiporn.th@cmu.ac.th (P.D.); juthamas.i@cmu.ac.th (J.I.); chaiwat.b@cmu.ac.th (C.B.); athavudh.d@cmu.ac.th (A.D.); theerakorn.t@cmu.ac.th (T.T.); atikun.limsukon@cmu.ac.th (A.L.); pattraporn.t@cmu.ac.th (P.T.); nutchanok.n@cmu.ac.th (N.N.); konlawij.tr@cmu.ac.th (K.T.); chittrawadee.chit@cmu.ac.th (C.C.); chaicharn.p@cmu.ac.th (C.P.); 2 Neurophysiology Unit, Cardiac Electrophysiology Research and Training Center, Faculty of Medicine, Chiang Mai University, Chiang Mai 50200, Thailand; nipon.chat@cmu.ac.th (N.C.); siriporn.c@cmu.ac.th (S.C.C.); nattayaporn.a@cmu.ac.th (N.A.); 3 Cardiac Electrophysiology Unit, Department of Physiology, Faculty of Medicine, Chiang Mai University, Chiang Mai 50200, Thailand; 4 Center of Excellence in Cardiac Electrophysiology, Chiang Mai University, Chiang Mai 50200, Thailand; 5 Department of Oral Biology and Diagnostic Sciences, Faculty of Dentistry, Chiang Mai University, Chiang Mai 50200, Thailand

**Keywords:** elderly COPD, healthy elderly, PM_2.5_, inflammatory biomarkers

## Abstract

Older adults and individuals with chronic obstructive pulmonary disease (COPD) are vulnerable to the harmful health effects of fine particulate matter (PM_2.5_), which are partly driven by systemic inflammation. This study examined whether older adults with COPD exhibit greater inflammatory responses to PM_2.5_ than age-matched healthy controls. We compared circulating high-sensitivity C-reactive protein (hsCRP), interleukin (IL)-6, IL-8, and tumor necrosis factor (TNF)-alpha in 38 patients with COPD (73.6 ± 7.1 years) and 20 controls (70.9 ± 5.2 years) during high- and low-pollution periods. In the COPD group, 65.8% used inhaled corticosteroids and 42.1% used statins; 65.0% of controls used statins. Mean PM_2.5_ concentrations were higher in the high-pollution period than the low-pollution period (79.1 ± 23.3 vs 13.9 ± 3.6 µg/m^3^; *p* < 0.001). Across both periods, IL-8 was higher in COPD than in controls, without significant within-group increases during high pollution. These findings indicate higher baseline systemic inflammation in COPD, while pollution-associated increases may be attenuated by anti-inflammatory medications. However, this should be interpreted cautiously given the observational design.

## 1. Introduction

Fine particulate matter with an aerodynamic diameter smaller than 2.5 µm (PM_2.5_), the primary air pollutant in Chiang Mai, frequently rises above the World Health Organization (WHO) safety guideline between January and April each year [1]. High PM_2.5_ concentrations in Chiang Mai occur during periods of low humidity, similar to patterns observed in other tropical countries such as India [2]. However, polluted episodes in Chiang Mai coincide with higher temperatures, whereas those in India are associated with lower temperatures, potentially reflecting differences in geography and PM_2.5_ sources. The principal sources of particulate matter in this region are forest fires and the burning of post-harvest agricultural residues [3].

PM_2.5_ affects not only the respiratory system, the primary route of entry, but also multiple organ systems through particle translocation from the alveoli across the pulmonary capillary into the systemic circulation. The major mechanisms of PM_2.5_-induced adverse health effects are oxidative stress and inflammation [4]. Systemic inflammation has been demonstrated by increased circulating inflammatory biomarkers, including high-sensitivity C-reactive protein (hsCRP) [5], interleukin-6 (IL-6) [6], IL-8, and tumor necrosis factor (TNF)-alpha [7], after acute exposure to PM_2.5_. Therefore, systemic inflammation is a plausible mechanistic link between PM_2.5_ exposure and the development of various non-respiratory diseases.

The harmful health impacts of short-term PM_2.5_ exposure are well established in older adults, especially in those with pre-existing cardiopulmonary conditions [8]. Age-related immune dysregulation (immunosenescence) is associated with chronic inflammation and contributes to systemic, degenerative, and metabolic disorders in the elderly [9]. Immunosenescent cells with a senescence-associated secretory phenotype (SASP) increasingly release proinflammatory cytokines (e.g., TNF-alpha, IL-8, and IL-6), followed by higher circulating CRP levels in response to diverse stimuli [9,10]. This process results in persistent low-grade inflammation (“inflammaging”), which may prime exaggerated inflammatory responses to multiple triggers, including infection and air pollution, in older adults [10,11]. Beyond pre-existing diseases and age-related physiological decline, PM_2.5_ exposure may further promote immunosenescence and subsequently induce heightened inflammatory and oxidative responses in the elderly [12,13]. Altuwayjiri et al. showed that PM_2.5_ exposure was associated with higher systemic inflammatory biomarkers, including IL-6 and hsCRP, in older adults compared with healthy adolescents [14]. Further evidence of vulnerability in older adults is provided by reports of increasing PM-associated deaths in aging populations in Asia from 1990 to 2019 [13]. Moreover, mortality associated with acute PM_2.5_ exposure increases significantly in older adults for both non-accidental and cardiovascular causes [15].

Among respiratory diseases affecting aging populations, chronic obstructive pulmonary disease (COPD) appears particularly susceptible to PM_2.5_. COPD is characterized by persistent, not fully reversible airflow limitation, primarily resulting from chronic respiratory inflammation associated with long-term exposure to noxious agents, particularly tobacco smoke and air pollution [16]. In addition to respiratory manifestations, COPD is associated with multiple comorbidities, especially cardiovascular diseases that are linked to systemic inflammation [16]. A systematic review and meta-analysis by Su et al. found that COPD is accompanied by elevated levels of systemic inflammatory biomarkers, such as CRP, IL-6, and IL-8 [17]. These biomarkers are also associated with adverse outcomes in COPD [18]. Agustí et al. reported that COPD patients with persistent systemic inflammation over 1 year had higher exacerbation rates and mortality than those without systemic inflammation [19]. Therefore, evidence has supported that COPD patients have baseline chronic respiratory and systemic inflammation. With short-term PM_2.5_ exposure, acute-on-chronic respiratory and systemic inflammation increases the risk of morbidity and mortality in COPD patients [20]. Recent PM_2.5_ exposure is associated with worsening symptoms, poorer quality of life and lung function [21], exacerbations [22], emergency department visits [1], and mortality [23]. Epidemiologic data indicate that short-term PM-associated mortality is higher in COPD patients, particularly in those of advanced age than in non-COPD populations [24]. Notably, the increased mortality linked to PM_2.5_ exposure among people with COPD is not confined to respiratory causes and is often due to cardiovascular events [25]. Alexeeff et al. further demonstrated that chronic PM_2.5_ exposure among COPD patients was associated with a significantly increased risk of cardiovascular death [26].

Collectively, this evidence supports a central role for systemic inflammation in mediating health effects of PM_2.5_ exposure in both older adults and patients with COPD. Chen et al. reported that, compared with controls, COPD patients exposed to PM_2.5_ had higher serum IL-8 and TNF-α levels; however, the control group included both middle-aged and older participants [27]. Whether PM_2.5_ elicits greater systemic inflammatory responses in older adults with COPD than in older adults without COPD remains clinically relevant. Therefore, the primary objective of this study was to determine the effects of PM_2.5_ exposure on systemic inflammatory biomarkers (hsCRP, IL-6, IL-8, and TNF-alpha) in older COPD patients compared with healthy older adults. The secondary objective was to compare these biomarkers between high- and low-pollution periods in COPD patients.

## 2. Results

### 2.1. Subject Characteristics

**Figure 1:**
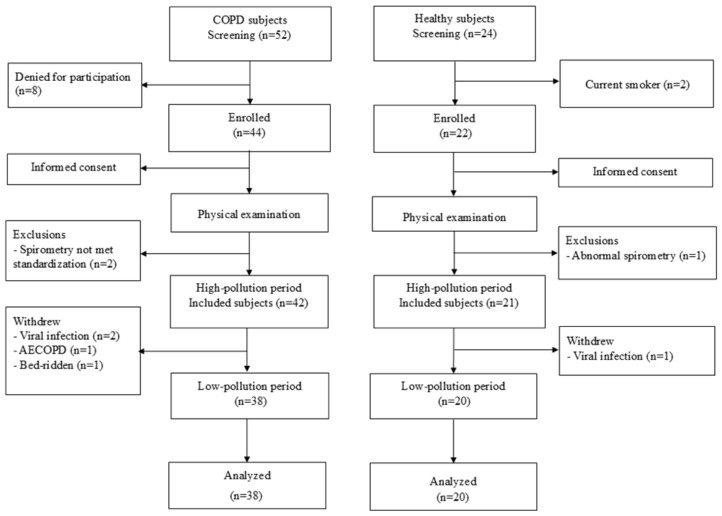
Study flow.

Fifty-two patients with COPD and twenty-four healthy older control subjects were screened. Ultimately, 38 COPD patients and 20 controls were included in the final analysis. Reasons for screening failure and participant withdrawal are presented in Figure 1. In the COPD group, four participants were lost to follow-up (one due to immobilization, one due to an AECOPD requiring mechanical ventilation, and two due to viral infection). In the control group, two participants were classified as screening failures because of current smoking, and two were withdrawn subsequently because of abnormal spirometry and development of viral infection prior to the scheduled visit. Of the two viral infections in the COPD group, one participant developed coronavirus disease 2019 (COVID-19) and one had an acute unidentified viral infection; in the control group, one participant was diagnosed with chikungunya prior to the follow-up visit.

No significant differences were observed between the COPD and healthy older adult groups with respect to age, proportion of males, body mass index (BMI), or educational level. The COPD group had a significantly higher proportion of ex-smokers and a greater smoking pack-year history than the control group. Information regarding exposure to polluted air during the pollution period including use of PM_2.5_ protective measures (e.g., N-95 mask), availability of a home air purifier, and duration of time spent in a room with an air purifier did not differ between groups. All COPD participants received inhaled maintenance therapy, 100% used long-acting bronchodilators, either LABA or LAMA, and 65.8% received ICS. Additional details are provided in Table 1.

### 2.2. Pollution Data, Temperature, and Humidity During Low- and High-Pollution Periods

Air pollution data for the low-pollution period (August 2023) and the high-pollution period (March 2023) are summarized in Table 2. The daily mean PM_2.5_ concentration during the high-pollution period was significantly higher than during the low-pollution period (79.1 ± 23.3 µg/m^3^ vs. 13.9 ± 3.6 µg/m^3^, *p* < 0.001). Daily mean temperature followed a similar trend, while the daily mean relative humidity was significantly lower during the high-pollution period. Accordingly, the high-pollution period occurred in summer and was characterized by significantly higher daily mean temperatures and lower relative humidity, whereas the low-pollution rainy period exhibited lower temperatures and higher relative humidity. Additional details are provided in Table 2.

### 2.3. Inflammatory Biomarkers in COPD Patients and Healthy Older Adults Across Low- and High-Pollution Periods (Table 3)

Median (IQR) hsCRP levels were comparable between the COPD and control groups in both the low-pollution period [2.4 (0.8, 4.8) mg/L vs. 1.0 (0.5, 2.7) mg/L] and the high-pollution period [1.8 (0.9, 3.5) mg/L vs. 0.8 (0.5, 2.3) mg/L]. Within-group comparisons showed no significant differences in hsCRP between the low- and high-pollution periods in both the COPD group (*p* = 0.140) and the control group (*p* = 0.776).

IL-6 levels were significantly higher in COPD than in controls during the low-pollution period [5.5 (3.2, 10.9) pg/mL vs. 3.2 (1.8, 4.9) pg/mL; *p* = 0.001], but did not differ between groups during the high-pollution period [4.9 (3.2, 6.8) pg/mL vs. 4.5 (1.9, 8.9) pg/mL; *p* = 0.621]. IL-6 did not differ between periods in the COPD group (*p* = 0.061).

IL-8 levels were significantly higher in COPD than in controls during both the low-pollution period [7.7 (5.3, 18.9) pg/mL vs. 4.0 (2.9, 7.9) pg/mL; *p* = 0.002] and the high-pollution period [8.5 (6.2, 19.2) pg/mL vs. 4.2 (3.3, 6.1) pg/mL; *p* = 0.001]. Within-group comparisons revealed no significant differences in IL-8 between the low- and high-pollution periods in either the COPD group (*p* = 0.455) or the control group (*p* = 0.852).

Median (IQR) TNF-alpha levels did not differ significantly between COPD and controls in the low-pollution period [18.7 (11.0, 21.6) pg/mL vs. 10.6 (8.6, 22.2) pg/mL; *p* = 0.256] or in the high-pollution period [19.0 (11.5, 21.8) pg/mL vs. 11.8 (8.1, 20.7) pg/mL; *p* = 0.157]. Likewise, within-group comparisons showed no significant differences in TNF-alpha between the low- and high-pollution periods in each group.

**Table 3:** Inflammatory biomarkers between the low-pollution and high-pollution periods in COPD and healthy elderly group.

Inflammatory Biomarkers	COPD (n = 38)			Healthy Elderly (n = 20)		
	Low-Pollution Period	High-Pollution Period	*p*-Value	Low-Pollution Period	High-Pollution Period	*p*-Value
hsCRP (mg/L)	2.4 (0.8, 4.8)	1.8 (0.9, 3.5)	0.140	1.0 (0.5, 2.7)	0.8 (0.5, 2.3)	0.776
IL-6 (pg/mL)	5.5 (3.2, 10.9) *	4.9 (3.2, 6.8)	0.061	3.2 (1.8, 4.9)	4.5 (1.9, 8.9)	0.042
IL-8 (pg/mL)	7.7 (5.3, 18.9) *	8.5 (6.2, 19.2) **	0.455	4.0 (2.9, 7.9)	4.2 (3.3, 6.1)	0.852
TNF-alpha (pg/mL)	18.7 (11.0, 21.6)	19.0 (11.5, 21.8)	0.934	10.6 (8.6, 22.2)	11.8 (8.1, 20.7)	0.687

**Notes:** Results are expressed as median (IQR); *, *p* < 0.0125 for comparison between groups in low-pollution period; **, *p* < 0.0125 for comparison between groups in high-pollution period. **Abbreviations:** COPD, chronic obstructive pulmonary disease; hsCRP, high-sensitivity C-reactive protein; IL-6, interleukin-6; IL-8, interleukin-8; TNF, tumor necrosis factor.

No significant differences were observed in systemic inflammatory biomarkers (hsCRP, IL-6, IL-8, and TNF-α) between medication users and non-users in either the COPD or control group during both the low- and high-pollution periods. Moreover, within-group comparisons between the low- and high-pollution periods, stratified by medication status, also showed no statistically significant changes. Additional details are provided in Supplementary Table S1.

## 3. Discussion

Inflammation and oxidative stress play key roles in COPD pathogenesis and in the deleterious health effects linked to PM_2.5_ exposure [28]. Systemic inflammation observed in both conditions may arise from comorbid diseases, spillover of inflammatory mediators from the respiratory tract, and/or direct effects of inhaled particles that translocate into the circulation [28,29]. In response to cigarette smoke and other harmful particles, macrophages act as major effector cells, releasing pro-inflammatory cytokines such as TNF-α, IL-6, and IL-8. These mediators then drive the recruitment of inflammatory cells, especially neutrophils [30].

Our study corroborated baseline systemic inflammation in COPD, as reflected by significantly higher IL-6 and IL-8 levels than in older control subjects during the low-pollution period, consistent with findings reported by Sanchez-Azofra et al. [31]. In contrast to our study, Sanchez-Azofra et al. included young and middle-aged adults as controls. In relation to our primary objective, after exposure to higher PM_2.5_ levels, between-group differences remained significant for IL-8 only.

Regarding IL-8, Wang et al. reported increases from baseline in both COPD and control groups following particulate matter exposure, without a significant difference between groups; however, their controls were not restricted to older adults and data on key confounders (e.g., comorbidities and medication use) were not available [32]. The PM_2.5_-related IL-8 findings in our study are more consistent with Chen et al., who observed significantly higher IL-8 levels in COPD patients than in controls [27]. However, unlike our results, Chen et al. also reported higher TNF-alpha levels in COPD patients than in controls during short-term PM_2.5_ exposure [27]. In our cohort, TNF-alpha did not differ significantly either between groups or within groups across pollution periods, which contrasts with several prior reports [6,27,32,33,34]. Notably, heterogeneous responses across the four biomarkers in COPD patients exposed to PM_2.5_ are in keeping with the systematic review by Kim et al. [35]. Such variability likely reflects differences in study design, COPD etiology (smoking-related vs non-smoking-related), smoking status, COPD severity, comorbidity profiles (e.g., obesity, dyslipidemia, diabetes, cardiovascular disease), concomitant medications for COPD and comorbidities, meteorological conditions (e.g., humidity and temperature), and PM_2.5_ exposure characteristics (e.g., ambient and indoor concentrations, sources, and chemical components).

In our study, the COPD group exhibited higher baseline IL-6 levels during the low-pollution period, in line with the findings of Singh et al. [33]. However, after PM_2.5_ exposure, IL-6 no longer differed significantly between COPD and controls. Regarding our secondary objective, an important finding was that IL-6 did not increase in COPD patients after exposure to high PM_2.5_ levels. Although this is consistent with Dadvand et al., who reported that short-term PM_2.5_ exposure did not increase IL-6 in COPD patients [36], most studies have reported the opposite [34]. Moreover, none of the biomarkers evaluated in our study increased in COPD patients from the pre-exposure to the high-exposure period. This contrasts with findings from Liu et al., who reported increases in CRP and TNF-alpha with PM_2.5_ exposure [37]. A plausible explanation for the lack of biomarker elevation in our COPD cohort is the influence of medications with antioxidant and/or anti-inflammatory properties [9]. In our study, 25 (65.8%) COPD patients received ICS, an important anti-inflammatory add-on therapy in COPD. ICS inhibit macrophage release of IL-6 and TNF-alpha, whereas suppression of IL-8 is relatively limited [38]. This medication profile may partly explain why IL-8 stayed significantly higher in COPD patients than in controls, while IL-6 and TNF-α did not show the same pattern.

Statins also have anti-inflammatory properties beyond lipid lowering and may attenuate systemic inflammation. Meta-analyses suggest that statins can improve exercise performance, COPD Assessment Test (CAT) scores, and pulmonary function in COPD [39]. In pulmonary hypertension-associated COPD (PH-COPD), adding statins to standard therapy may improve exercise capacity, lung function, oxygenation, pulmonary arterial pressure, and inflammatory biomarkers, including TNF-alpha, IL-6, and hsCRP [40]. Statins have also been shown to reduce TNF-alpha [41] and CRP [42] in patients with cardiovascular disease. In older adults exposed to high PM_2.5_, statin use has been associated with reduced cardiovascular risk [43], including stroke [44], and cardiovascular mortality [45]. Several studies also indicate that, among COPD patients, particulate matter exposure is linked to smaller rises in CRP and IL-6 in those taking statins compared with those not treated with statins [46,47]. Moreover, substantial evidence shows that statin use is associated with lower levels of CRP, TNF-α, IL-6, and IL-8 in chronic inflammatory conditions [48,49]. Because nearly half of the COPD patients and about two-thirds of the controls in our study were taking statins, their anti-inflammatory effects may help explain why biomarker levels did not rise after PM_2.5_ exposure in either group.

Other medications that may influence biomarker levels in the COPD group include metformin and N-acetyl cysteine (NAC). Beyond its glucose-lowering action, metformin exerts anti-inflammatory effects, largely by activating the 5′-adenosine monophosphate-activated protein kinase (AMPK) pathway [50]. Multiple studies have reported reductions in TNF-alpha, IL-6, and CRP with metformin, although the results are inconsistent, likely due to differences in study populations and designs [50]. Although only five (13.2%) COPD patients received metformin, its effects in combination with other anti-inflammatory or antioxidant therapies may have contributed to biomarker variability.

NAC, a mucolytic with antioxidant activity, may dampen inflammation driven by oxidative stress. Although long-term, high-dose NAC did not lower the annual rate of severe acute exacerbations of COPD (AECOPD), it did significantly reduce moderate AECOPD [51], particularly among patients with forced expiratory volume in the first second (FEV_1_) > 50% predicted [52]. Animal data also suggest a protective effect of NAC against TNF-alpha and IL-6 production following acute PM_2.5_ exposure [53]. Although only 11 (28.9%) COPD participants used NAC, it may blunt biomarker increases in response to PM_2.5_.

One possible explanation for the absence of significant increases in IL-6 (and other systemic inflammatory biomarkers) among COPD participants during the high-PM_2.5_ period is a “ceiling effect.” In this scenario, COPD is associated with chronically elevated systemic inflammation, which may limit the extent to which inflammatory markers can increase further in response to short-term PM_2.5_ exposure. By contrast, healthy older adults typically have lower and less variable baseline inflammation and may therefore show a more detectable, exposure-induced increase in cytokines such as IL-6 when PM_2.5_ levels are high. This interpretation is also consistent with other factors that may mask small exposure-related changes, including variability in COPD severity, the timing of blood sampling in relation to exposure, and the use of anti-inflammatory treatments. However, because this was an observational pilot study with limited statistical power, we cannot determine whether the observed pattern reflects true biological saturation or instead results from variability or limited sensitivity to detect modest effects. Larger studies with repeated biomarker measurements and more detailed exposure assessments are needed to test this hypothesis.

A key strength of our study is that, to our knowledge, it is the first to directly compare systemic inflammatory biomarker responses to PM_2.5_ exposure between two high-risk groups. Moreover, we restricted enrollment to older adults and excluded obesity and current smoking, two factors that can substantially confound inflammatory biomarker levels. We also assessed biomarkers across two periods with distinct PM_2.5_ exposure levels to evaluate both baseline and post-exposure concentrations in each group. Nevertheless, several limitations should be acknowledged. First, we did not control dietary or supplement intake of antioxidant and anti-inflammatory nutrients (e.g., fruits, vegetables, vitamin C, vitamin E, carotenoids, polyphenols, and omega-3 fatty acids), which may influence systemic inflammation. Wang et al. reported that higher vegetable consumption was associated with a lower risk of PM_2.5_-associated cardiovascular mortality [54]. Parklak et al. further showed that higher dietary fiber and antioxidant vitamin intake lowered IL-6 and CRP during high PM_2.5_ exposure in healthy volunteers [55]. Additionally, use of concomitant anti-inflammatory medications, particularly nonsteroidal anti-inflammatory drugs (NSAIDs) and acetylsalicylic acid (aspirin), was not systematically assessed and may have introduced residual confounding in inflammatory biomarker analyses. Future studies should collect detailed NSAIDs and aspirin exposure and adjust and/or stratify analyses accordingly. Second, though not statistically significant, air purifier use was more common among controls and may have attenuated their effective PM_2.5_ exposure and biomarker responses; we did not standardize or objectively verify PM_2.5_ protective measures (use of N-95 masks, availability and duration of air purifier use), including assessment of correct N-95 use (fit testing and seal checks), nor did we measure indoor PM_2.5_ reductions after air purifier use; these factors may have introduced exposure misclassification and confounding. Third, the study was the pilot study. The subsequent studies which include more subjects are required. Fourth, this study was not designed to quantify the effects of anti-inflammatory and antioxidant medications on biomarker levels. Thus, the hypothesis that these agents mitigate PM_2.5_-induced cytokine responses in older adults with and without COPD requires confirmation in future studies. Fifth, this observational study is that the COPD and healthy elderly groups have inherently different baseline inflammatory profiles, which may confound disease-related inflammation with PM_2.5_-induced systemic inflammatory responses. Regarding comorbidities, although a larger proportion of healthy controls had cardiovascular disease, this difference was not statistically significant and was not included as a confounder in our analysis. Nevertheless, cardiovascular comorbidity may influence inflammatory cytokine expression, and residual confounding from this factor cannot be excluded. Sixth, we did not collect baseline data for screen-failure participants; therefore, we were unable to compare characteristics between completers and non-completers, and the small number of withdrawals/exclusions (14 COPD and 4 healthy elderly) further limited the statistical power to assess potential attrition bias. Seventh, since seasonal meteorology exerts complex influences on PM_2.5_ dynamics, the low- and high-exposure periods occurred in different seasons and likely differed in aerosol composition and other covariate seasonal factors (e.g., temperature, humidity, wind speed, and direction, and circulating respiratory infections) [2]. Because aerosol composition was not measured and season was not included as a confounder, residual seasonal confounding cannot be excluded. Moreover, we did not evaluate delayed (lagged) effects of PM_2.5_ exposure (e.g., over the subsequent 1–7 days) on systemic inflammatory biomarkers, which might lead to underestimation of pollution-related inflammatory responses. Additionally, because of the limited pilot sample size, we did not perform exposure–response correlation analyses or multivariable logistic regression modeling. Therefore, we acknowledge this as a limitation that should be addressed in larger future studies with improved individual-level exposure assessment. Lastly, the generalizability of these findings beyond Chiang Mai may be limited, as regional disparities in PM_2.5_ sources, chemical compositions, meteorological profiles, healthcare practices (including medication usage), and underlying population susceptibilities can lead to distinct biomarker profiles and exposure–response patterns.

## 4. Materials and Methods

### 4.1. Study Design and Population

A prospective observational study was conducted during 17–31 March 2023 (high-pollution period) and 9–18 August 2023 (low-pollution period). Out-patients with COPD were enrolled at the Lung Health Center, Division of Pulmonary, Critical Care, and Allergy, Department of Internal Medicine, Faculty of Medicine, Chiang Mai University, Chiang Mai, Thailand. Healthy controls with age > 60 years were recruited from the community via poster advertisements. All participants resided in Chiang Mai Province, and the same individuals were evaluated in both the low- and high-PM_2.5_ periods. Patients with COPD were required to meet all of the following inclusion criteria: age > 60 years; a diagnosis of COPD based on a post-bronchodilator (BD) FEV_1_/forced vital capacity (FVC) ratio < 0.7, in accordance with the Global Initiative for Chronic Obstructive Lung Disease (GOLD) criteria [16]; ex-smoker for > 6 months with a smoking history > 10 pack-years; no acute exacerbation (AECOPD) for at least 3 months prior to enrollment; and receipt of long-term pharmacological COPD treatment with stable doses of inhaled corticosteroids (ICS), long-acting beta-2 agonists (LABA), and long-acting muscarinic antagonists (LAMA) for at least 3 months before enrollment. Exclusion criteria were: uncontrolled non-COPD comorbidities; a diagnosis of asthma or asthma–COPD overlap (ACO) established by a chest physician and documented in the medical record at any time, regardless of recent symptoms or exacerbations; active respiratory disorders other than COPD (e.g., lung cancer, tuberculosis, or bronchiectasis); body mass index (BMI) > 30 kg/m^2^; or other significant abnormal chest radiographic findings not attributable to COPD within the past year. Healthy older adults without history of any respiratory diseases, with a history of never smoking or being an ex-smoker for ≥6 months prior to enrollment, controlled comorbidities, and BMI < 30 kg/m^2^ were included as comparable controls. The study was approved by the Research Ethics Committee, Faculty of Medicine, Chiang Mai University (study code: MED-2565-009244; date of approval: 28 October 2022), and registered in the Clinical Trials Registry (study ID: TCTR20221118003; date of approval: 18 November 2022). Written informed consent was obtained from all participants prior to enrollment.

Baseline demographic and clinical data including age, sex, BMI, smoking history, comorbidities, medication use, and educational attainment were collected from the medical record. We also recorded air pollution protective practices, such as using an indoor air purifier and wearing an N-95 mask outdoors during the pollution season. Systemic inflammatory biomarkers (hsCRP, IL-6, IL-8, and TNF-α) were measured in both groups during periods of high and low pollution.

### 4.2. Measurements of PM_2.5_ and Meteorological Parameters

PM_2.5_ concentrations were obtained from monitoring stations of the Pollution Control Department, Ministry of National Resources and Environment, located in municipal areas of Muang Chiang Mai District, Chiang Mai Province, Thailand [56]. The cut-off to define high versus low daily average PM_2.5_ in this study was based on the 2021 WHO Air Quality Guidelines, using a 24 h mean PM_2.5_ level of 15 µg/m^3^ [57]. Daily ambient PM_2.5_ data were obtained for the days corresponding to participants’ study visits during the prespecified low- and high-exposure periods; the duration of each period therefore differed based on the visit schedule and participant attendance. There were two distinct tracking windows: a high-exposure period during the summer season (17–31 March 2023) and a low-exposure period during the rainy season (9–18 August 2023). For each period, PM_2.5_ exposure was summarized as the mean of the daily 24 h average PM_2.5_ concentrations across all included days. During the high-exposure period, none of the included days had a 24 h mean PM_2.5_ concentration below 15 µg/m^3^. Meteorological data (temperature and humidity) were obtained from the Northern Meteorology Center, Chiang Mai Province; the meteorological monitoring station was also located in the municipal area of Muang Chiang Mai District, Chiang Mai, Thailand.

### 4.3. Outcome Measures

#### Systemic Inflammatory Biomarkers

Venous blood was drawn from all participants to assess hsCRP, IL-6, IL-8, and TNF-α. Serum hsCRP was quantified using an immunoturbidimetric assay (Cobas^®^ pro-C503, Baku, Azerbaijan). Serum IL-6, IL-8, and TNF-α levels were measured with commercially available solid-phase sandwich ELISA kits (Invitrogen, Waltham, MA, USA; IL-6 Catalog no. BMS213-2HS, IL-8 Catalog no. KHC0084, and TNF-α Catalog no. BMS223HS). All procedures were conducted in accordance with the manufacturers’ protocols.

### 4.4. Statistical Analysis

Continuous variables are presented as mean ± standard deviation (SD) or median (interquartile range, IQR), as appropriate based on distribution. Categorical variables are presented as number and percentage (%). Baseline characteristics and systemic inflammatory biomarkers were compared between the COPD and control groups using an independent *t*-test for normally distributed data or the Mann–Whitney U test for non-normally distributed data. Fisher’s exact test was used to compare categorical variables between groups. Pollutant levels between the low- and high-pollution periods were compared using an independent *t*-test. Within-group comparisons of inflammatory biomarkers between low- and high-pollution periods were performed using a paired *t*-test or the Wilcoxon signed-rank test, as appropriate. As an exploratory analysis, we assessed whether changes in systemic inflammatory biomarkers from the low- to high-pollution period differed by medication status by comparing biomarker levels between medication users and non-users using the Mann–Whitney U test and evaluating within-group low–high period differences separately within each medication stratum using the Wilcoxon signed-rank test (in both COPD and control groups). Statistical significance was defined as *p* < 0.05. To account for multiple comparisons, analyses were conducted separately for each biomarker across the two groups (COPD vs. healthy elderly) and two exposure periods (low vs. high pollution periods), resulting in four planned comparisons per biomarker. Therefore, a Bonferroni correction was applied within each biomarker, and statistical significance was defined as a two-sided *p*-value < 0.0125 (0.05/4). All tests were two-tailed, and adjusted significance levels were used when interpreting results. All analyses were performed using STATA version 16 (StataCorp, College Station, TX, USA).

## 5. Conclusions

This study provides evidence of systemic inflammation in both older adults with COPD and healthy older adults in relation to PM_2.5_ exposure. Baseline inflammatory biomarkers were significantly higher in COPD than in older controls. During high PM_2.5_ exposure, IL-8 remained significantly higher in COPD than in controls. Biomarkers did not increase from the low- to high-pollution period in COPD patients or healthy older adults. Medications with anti-inflammatory activity, particularly ICS and statins, may have influenced biomarker levels. In addition to minimizing PM_2.5_ exposure, adherence to therapies, especially those with anti-inflammatory effects, used to manage underlying diseases may help mitigate PM_2.5_-related health risks. However, this hypothesis cannot be confirmed in this observational pilot study.

## Figures and Tables

**Table 1:** Demographic data of study participants and information on exposure to polluted air in COPD and healthy elderly group.

Variables	COPD (n = 38)	Healthy Elderly (n = 20)	*p*-Value
Age (years)	73.6 ± 7.1	70.9 ± 5.2	0.142
Male sex, n (%)	34 (89.5)	17 (85.0)	0.683
Height (cm)	159.7 ± 7.7	159.9 ± 7.7	0.911
Body weight (kg)	56.9 ± 13.3	61.6 ± 8.7	0.162
BMI (kg/m^2^)	22.2 ± 4.1	24.1 ± 2.9	0.070
Smoking status n (%)			<0.001
Non-smoker	3 (7.9)	13 (65.0)	
Current smoker	0 (0.0)	0 (0.0)	
Ex-smoker	35 (92.1)	7 (35.0)	
Smoking pack-year, median (IQR)	24.6 (11.2, 40.0)	0.0 (0.0, 5.0)	<0.001
Education level			0.088
Primary	21 (55.3)	5 (25.0)	
Secondary	6 (15.8)	5 (25.0)	
Bachelor’s degree or higher	11 (28.9)	10 (50.0)	
Comorbid diseases			0.163
No	15 (39.5)	7 (35.0)	
Cardiovascular disease only	14 (36.8)	12 (60.0)	
Diabetes mellitus only	1 (2.6)	1 (5.0)	
Both cardiovascular disease and diabetes mellitus	8 (21.1)	0 (0.0)	
Inhaled medication used			
LAMA	6 (15.8)	N.A.	
LABA + LAMA	7 (18.4)	N.A.	
ICS + LABA	7 (18.4)	N.A.	
ICS + LABA + LAMA	18 (47.4)	N.A.	
N-acetyl cysteine (NAC) use	11 (28.9)	N.A.	
Statins use	16 (42.1)	13 (65.0)	0.167
Metformin use	5 (13.2%)	0 (0.0)	0.153
**Information on Exposure to Polluted Air in Pollution Period**			
Wearing PM_2.5_ protection mask, i.e., N-95 mask (yes)	8 (21.1)	5 (25.0)	0.750
Having an air purifier * in the house (yes)	17 (44.7)	12 (60.0)	0.408
Duration of staying in the room with air purifier * (hours/day), median (IQR)	10.0 (10.0, 20.0)	10.0 (3.5, 18.0)	0.325

**Notes:** Results are expressed as mean ± SD, n (%), median with interquartile range (IQR) or otherwise stated. * Air purifier with a high-efficiency particulate air (HEPA) filter. **Abbreviations:** BMI, body mass index; COPD, chronic obstructive pulmonary disease; ICS, inhaled corticosteroids; LABA, long-acting beta 2-agonist; LAMA, long-acting muscarinic antagonist; N.A., not available.

**Table 2:** Daily average of PM_2.5_, temperature, and humidity between the high-pollution period (March 2023) and the low-pollution period (August 2023).

Variables	Low-Pollution Period	High-Pollution Period	*p*-Value
PM_2.5_ (µg/m^3^)	13.9 ± 3.6	79.1 ± 23.3	<0.001
Temperature (Celsius)	25.6 ± 1.8	27.4 ± 1.6	<0.001
Humidity (%)	78.1 ± 10.0	56.5 ± 7.3	<0.001

**Note:** Results are expressed as mean ± SD. **Abbreviations:** PM_2.5_, particulate matter with a diameter of smaller than 2.5 microns; m^3^, cubic meter.

## Data Availability

The data that support the findings of this study are available from the corresponding author upon reasonable request.

## Supplementary Materials

The following supporting information can be downloaded at: https://www.mdpi.com/article/10.3390/ijms27146283/s1.
