# Supplementary material for: Effects of Fine Particulate Matter on Systemic Inflammatory Biomarkers in Elderly Patients with Chronic Obstructive Pulmonary Disease Versus the Healthy Elderly: A Pilot Study
Source: Int J Mol Sci. 2026 Jul 15;27(14):6283. doi: 10.3390/ijms27146283 (PMC13409774; doi:10.3390/ijms27146283)
Supplement: Supplementary file 1 [file ijms-27-06283-s001.zip › ijms-4420066-supplementary.pdf]

Supplementary Table S1. Inflammatory Biomarkers between Users and Non-Users of Medications with Anti-Inflammatory properties (Any of Inhaled Corticosteroids, Statins, or N-Acetyl Cysteine) during Pollution and Non-Pollution Periods

Variables	Pollution period			Non-pollution period		
	**Medication users (n=32)**	**Non-medication users (n=6)**	**p-value**	**Medication users (n=32)**	**Non-medication users (n=6)**	**p-value**
**COPD (n=38)**						
hsCRP (mg/L)	1.9 (0.8, 3.7)	1.3 (0.9, 8.3)	0.873	2.4 (0.8, 4.1)	5.9 (0.6, 44.4)	0.536
IL-6 (pg/ml)	4.9 (3.5, 6.8)	4.9 (2.0, 9.1)	0.682	4.9 (3.2, 8.5)	12.0 (5.8, 26.0)	0.022
IL-8 (pg/ml)	8.9 (5.9, 17.3)	7.6 (5.8, 24.1)	0.841	7.7 (5.3, 16.9)	10.7 (4.4, 21.6)	0.968
TNF-alpha (pg/ml)	19.0 (11.5, 22.3)	18.1 (11.0, 21.8)	0.826	18.8 (11.2, 26.3)	17.4 (9.6, 31.8)	0.904
**Healthy elderly (n=20)**	**Medication users (n=8)**	**Non-medication users (n=12)**	**p-value**	**Medication users (n=8)**	**Non-medication users (n=12)**	**p-value**
hsCRP (mg/L)	0.8 (0.4, 2.9)	0.8 (0.5, 2.3)	0.877	0.8 (0.4, 3.1)	1.2 (0.6, 2.4)	0.727
IL-6 (pg/ml)	4.5 (1.9, 6.2)	6.8 (2.0, 10.5)	0.461	2.6 (1.7, 6.9)	3.2 (2.1, 4.9)	0.846
IL-8 (pg/ml)	3.5 (3.0, 4.3)	5.3 (3.5, 8.2)	0.090	3.2 (2.9, 3.9)	5.9 (3.6, 11.7)	0.076
TNF-alpha (pg/ml)	10.8 (8.0, 18.8)	17.9 (8.1, 21.9)	0.700	10.4 (6.9, 20.8)	18.4 (9.1, 22.2)	0.512

Notes: Results are expressed as mean ± SD, median (IQR).
Abbreviations: COPD, chronic obstructive pulmonary disease; hsCRP, high sensitivity C-reactive protein; IL-6, interleukin-6; IL-8, interleukin 8; TNF, tumor necrosis factor.
